# Elevated Plasma CXCL12α Is Associated with a Poorer Prognosis in Pulmonary Arterial Hypertension

**DOI:** 10.1371/journal.pone.0123709

**Published:** 2015-04-09

**Authors:** Brian N. McCullagh, Christine M. Costello, Lili Li, Caroline O’Connell, Mary Codd, Allan Lawrie, Allison Morton, David G. Kiely, Robin Condliffe, Charles Elliot, Paul McLoughlin, Sean Gaine

**Affiliations:** 1 School of Medicine and Medical Science, UCD Conway Institute, University College Dublin, Dublin 4, Ireland; 2 School of Public Health, Physiotherapy and Population Science, University College Dublin, Dublin 4, Ireland; 3 Department of Cardiovascular Sciences, University of Sheffield, Sheffield, United Kingdom; 4 Department of Cardiology, Northern General Hospital, Sheffield, United Kingdom; 5 Sheffield Pulmonary Vascular Disease Unit, Royal Hallamshire Hospital, Sheffield, United Kingdom; 6 Pulmonary Hypertension Unit, Mater Misericordiae University Hospital, Dublin 7, Ireland; University of Pécs Medical School, HUNGARY

## Abstract

**Rationale:**

Recent work in preclinical models suggests that signalling via the pro-angiogenic and pro-inflammatory cytokine, CXCL12 (SDF-1), plays an important pathogenic role in pulmonary hypertension (PH). The objective of this study was to establish whether circulating concentrations of CXCL12α were elevated in patients with PAH and related to mortality.

**Methods:**

Plasma samples were collected from patients with idiopathic pulmonary arterial hypertension (IPAH) and PAH associated with connective tissue diseases (CTD-PAH) attending two pulmonary hypertension referral centres (n = 95) and from age and gender matched healthy controls (n = 44). Patients were subsequently monitored throughout a period of five years.

**Results:**

CXCL12α concentrations were elevated in PAH groups compared to controls (P<0.05) and receiver-operating-characteristic analysis showed that plasma CXCL12α concentrations discriminated patients from healthy controls (AUC 0.80, 95% confidence interval 0.73-0.88). Kaplan Meier analysis indicated that elevated plasma CXCL12α concentration was associated with reduced survival (P<0.01). Multivariate Cox proportional hazards model showed that elevated CXCL12α independently predicted (P<0.05) earlier death in PAH with a hazard ratio (95% confidence interval) of 2.25 (1.01-5.00). In the largest subset by WHO functional class (Class 3, 65% of patients) elevated CXCL12α independently predicted (P<0.05) earlier death, hazard ratio 2.27 (1.05-4.89).

**Conclusions:**

Our data show that elevated concentrations of circulating CXCL12α in PAH predicted poorer survival. Furthermore, elevated circulating CXCL12α was an independent risk factor for death that could potentially be included in a prognostic model and guide therapy.

## Introduction

Pulmonary arterial hypertension (PAH) is characterized by increased pulmonary vascular resistance, mediated by structural remodelling of the pulmonary vasculature, endothelial dysfunction and inflammation [1–3]. These changes ultimately lead to right ventricular dysfunction, which is associated with higher morbidity and mortality [4]. The precise molecular mechanisms that cause these vascular abnormalities remain to be fully elucidated. In a previous study identifying genes selectively upregulated in the hypoxic lung we showed that CXCR7, a receptor for the pro-angiogenic chemokine CXCL12 (SDF-1), was selectively up-regulated in primary human pulmonary microvascular endothelial cells in response to hypoxia but remained unchanged in hypoxic systemic microvascular cells [5]. This selective increase in expression was also observed *in vivo* in the hypoxic rodent lung and in the endothelium of explanted human lungs from idiopathic (IPAH) patients [6]. We and others have also observed that both CXCL12 and a second CXCL12 receptor, CXCR4, were more highly expressed in remodelled vessels in hypertensive diseases compared to control lungs [6, 7]. Subsequently, the important role of CXCL12 in hypoxic PH was established by studies showing that inhibition of CXCL12 signalling via either of its two receptors, CXCR4 or CXCR7, attenuates hypoxia-induced PH in rodents [8–12]. Given these findings, the objective of the present study was to establish whether circulating concentrations of CXCL12α were increased in the plasma of patients with pulmonary hypertension and whether elevated concentrations predicted a poorer prognosis. Results from our study suggest that elevated circulating CXCL12α is an independent risk factor for reduced survival.

## Material and Methods

### Dublin patients and ELISA analysis

All patients attending the national pulmonary hypertension unit in the Mater Misericordiae University Hospital (MMUH), a referral centre for the assessment and treatment of PAH, between 7^th^ March 2007 and 22^nd^ July 2010 were recruited to the study. The study protocol was approved by the Mater Misericordiae Hospital ethics research committee and informed written consent was obtained from all study participants. BMPR2 mutation status was not ascertained. All patients (n = 43) had a diagnosis of PH confirmed by right heart catheterization demonstrating a mean pulmonary arterial pressure (mPAP) greater than 25 mmHg together with a pulmonary capillary wedge pressure less than or equal to 15 mmHg. Patients were recruited who had a diagnosis of PAH (Group 1) based on the Nice classification 2013 [3]; these were patients with idiopathic PAH (IPAH; Group 1.1) or connective tissue disease PAH (CTD-PAH, Group 1.4.1). Patient characteristics were recorded within 30 days of recruitment into the study from the clinical notes; more detailed information on patient diagnosis is available in S1 File. Eight patients were treatment naïve (incident disease cohort) whereas the remaining 35 patients (prevalent disease cohort) were receiving specific PAH therapy (Table 1). Survival status for the Dublin cohort was finally ascertained on 31^st^ July 2012 (i.e. 63-month follow-up period) by later clinical review, communication with the referring physician or checking the registry of deaths. Since many deaths occurred distant from the pulmonary hypertension centre, all-cause mortality was recorded to avoid bias in ascertaining the cause of death; one CTD patient was lost to follow-up. The Dublin cohort included a sub-group of patients (n = 12) included in our previous study and subsequently followed clinically for an extended period [6]. Venous blood samples were drawn, anti-coagulated and the plasma separated by centrifugation. Plasma samples were divided into aliquots and stored at −80°C before analysis. CXCL12α concentrations in undiluted plasma samples were measured by ELISA (DSA00—R&D Systems) in Dublin.

**Table 1:** Patient characteristics for Dublin (n = 43) and Sheffield cohorts (n = 52) at time of diagnostic catheter study.

		Dublin Cohort		Sheffield Cohort	
Characteristic		IPAH	CTD-PAH	IPAH	CTD-PAH
**Age (yrs)**		48 (38–59)	62 (49–71)	64 (44–68)	64 (61–70)
**Gender (F:M)**		16:5	18:4	14:11	17:10
**Ethnicity**	Caucasian	19	22	21	27
	Asian	2	-	4	-
**mPAP (mmHg)**		55 (42–61)	41 (28–50)	53 (49–63)	38 (30–46)
**PCWP (mmHg)**		10 (8–12)	10 (7–12)	11 (8–12)	12 (8–13)
**mRAP (mmHg)**		7 (5–10)	7 (4–8)	11 (8–15)	9 (4–10)
**PVR, dyn.s/cm** ^**5**^		864 (446–1085)	431 (332–738)	793 (539–1200)	412 (279–570)
**WHO FC**	I	1	1	0	0
	II	11	5	3	4
	III	7	14	21	20
	IV	2	2	1	3
**6MWT distance (m)**		425 (313–478)	375 (285–442)	-	-
**ISWT distance (m)**		-	-	190 (70–263)	110 (70–205)
**PAH therapies**	None	2	6	25	27
	ERA	16	11	0	0
	PDE5 inhibitor	9	6	0	0
	Prostacyclin analogues	7	4	0	0

Data presented as median (quartiles). Walk distance was measured by the 6-minute walk test (6MWT) in Dublin and the incremental shuttle walk test (ISWT) in Sheffield. One patient from Dublin was subsequently lost to follow-up (CTD patient, WHO-FC 1).

Definition of abbreviations: IPAH = Idiopathic pulmonary arterial hypertension, CTD = Connective tissue disease, mPAP = Mean Pulmonary Artery Pressure, mRAP = Mean Right Atrial Pressure, PCWP = Pulmonary Capillary Wedge Pressure, PVR = pulmonary vascular resistance, WHO FC = World health organization functional class, ERA = Endothelin receptor antagonist, PDE5 = Phosphodiesterase-5 inhibitor.

### Sheffield patients and ELISA analysis

Plasma samples were obtained from all patients referred to the pulmonary vascular disease unit in the Royal Hallamshire Hospital (Sheffield, UK), a referral centre for the assessment and treatment of PAH, between 28^th^ September 2009 and 17^th^ November 2011, as previously described [13]. Samples were obtained with Royal Hallamshire Hospital research ethics committee approval and informed written consent was obtained from all subjects. BMPR2 mutation status was not ascertained. Patients (n = 52) who had a diagnosis of IPAH or CTD-PAH, based on the criteria outlined above for the Dublin Cohort, were included in the study and had not received specific treatment for PH (included in the incident disease cohort) prior to the date of plasma sampling (Table 1). Venous blood was anti-coagulated and the plasma separated by centrifugation. Within 24 hours of blood sampling the age, gender and results of clinical assessment were recorded as for the Dublin Cohort, with the exception of the results of an incremental shuttle walk test which was used instead of the 6-minute walk test used in Dublin. Survival status was finally ascertained on 20^th^ June 2014 (i.e. 57-month follow-up period) by later clinical review, communication with the referring physician or checking the registry of deaths. Since many deaths occurred distant from the pulmonary hypertension centre, all-cause mortality was recorded to avoid bias in ascertaining the cause of death; no patient was lost to follow-up. Plasma samples were shipped to Dublin where CXCL12α concentrations in undiluted plasma samples were measured by ELISA (DSA00—R&D Systems) according to the manufacturer’s protocol.

### Control cohort and interassay controls

A panel of healthy non-smoking controls (n = 44), with no history of lung disease, was recruited from unaffected relatives of patients or unaffected relatives and colleagues of scientists in Dublin and processed as described above for the Dublin cohort. Undiluted plasma samples were measured by ELISA (DSA00—R&D Systems) in Dublin so that for each patient in each of the separate patient groups (IPAH-Dublin, CTD-PAH Dublin, IPAH-Sheffield, CTD-PAH Sheffield), a plasma sample could be obtained from a normal subject whose age and gender matched that of the patient. Four PAH plasma samples were assessed on all plates to act as interassay controls. For detailed methods, see S1 File.

### Statistical analyses

CXCL12α concentrations are presented as scatter-plots or medians (quartiles) and statistical comparisons made using the Mann-Whitney U test. The relationship between patient characteristics and biomarkers was assessed by Spearman rank test, or Chi-Square test for categorical variables. Receiver operating characteristic (ROC) curves were used to examine the usefulness of circulating CXCL12α concentrations in discriminating patients with PAH from healthy controls. All Kaplan-Meier analyses were performed using time from sampling to death/census, as assessed by the log-rank (Mantel-Cox) test. Univariate and multivariate analyses were conducted using Cox proportional hazards model with survival as the dependent variable. Independent variables, collected at the time of plasma sampling in both Dublin and Sheffield and entered into the analysis, were those identified in previous studies as of potential prognostic importance i.e. gender, age, WHO functional class, disease aetiology and interval from diagnosis to study enrolment (prevalent or incident disease) [14, 15]. There were no missing data in any of the variables included in the Cox Proportional analyses. Selected variables were input into a multivariable Cox proportional hazards model to identify independent prognostic factors; the model was fit with a p<0.10 entry criteria. All statistical analyses were performed with SPSS statistics version 20 software (IBM, USA); a P value less than 0.05 was considered statistically significant.

## Results

### CXCL12α concentrations in patient cohorts

**Fig 1:**
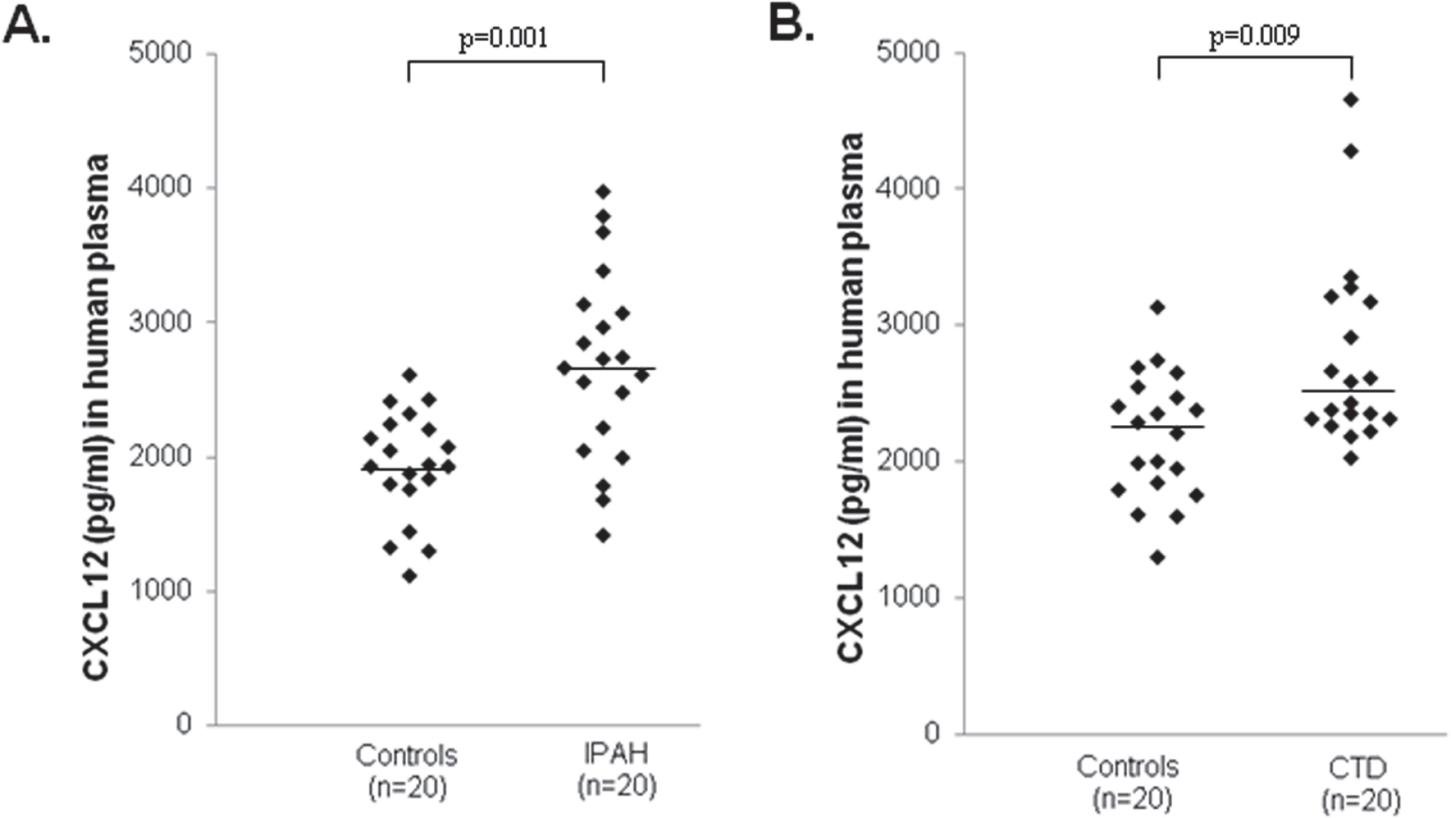
Plasma CXCL12α concentrations in pulmonary hypertensive patients (Dublin cohort) and age and gender matched controls. CXCL12α concentrations were significantly elevated in plasma samples from (A) idiopathic pulmonary arterial hypertensive (IPAH) patients (median: 2700, quartiles: 2174–3085 pg/ml) compared to age and gender matched controls (1938, 1784–2210 pg/ml) and from (B) connective tissue disease PAH (CTD-PAH) patients (2506, 2314–3180 pg/ml) compared to age and gender matched controls (2251, 1839–2488 pg/ml). Horizontal bars indicate median values.

**Fig 2:**
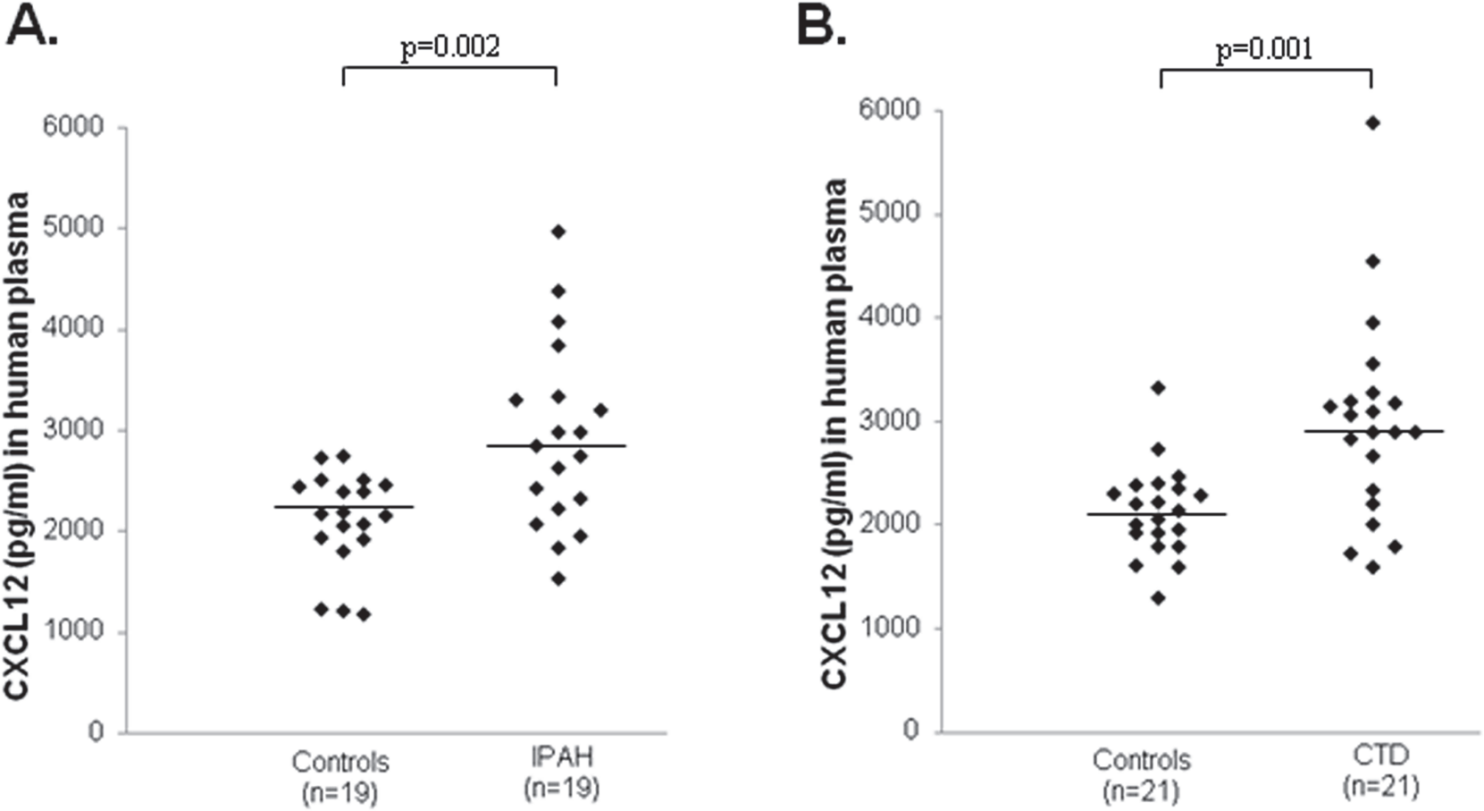
Plasma CXCL12α concentrations in pulmonary hypertensive patients (Sheffield cohort) and age and gender matched controls. CXCL12α concentrations were significantly elevated in plasma samples from (A) idiopathic pulmonary arterial hypertensive (IPAH) patients (median: 2845 pg/ml, quartiles: 2278–3320 pg/ml) compared to age and gender matched controls (2181, 1932–2457 pg/ml) and from (B) connective tissue disease PAH (CTD-PAH) patients (2903, 2332–3208 pg/ml) compared to age and gender matched controls (2143, 1924–2360 pg/ml). Horizontal bars indicate median values.

**Fig 3:**
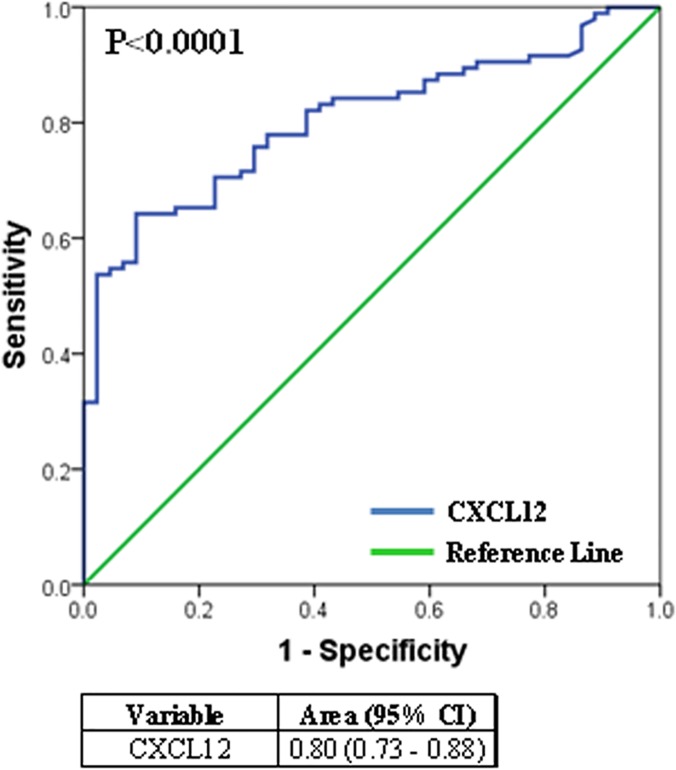
Receiver operating characteristic (ROC) analysis. Receiver operating characteristic (ROC) analysis showing the discrimination of healthy controls (n = 44) from PAH patients ((IPAH and CTD-PAH); n = 95) cohorts.

All ELISA analyses were carried out in Dublin, the ELISA kit (DSA00; R&D Systems) used allows the quantitative determination of human isoform CXCL12α. Patient characteristics for the Dublin group are reported in Table 1; one CTD patient (WHO-FC 1) was subsequently lost to follow-up due to emigration. CXCL12α concentrations were significantly elevated in patients with IPAH and CTD-PAH when compared to non-diseased age and gender matched controls (Fig 1 and Table A in S1 File). Given these findings, we decided to further explore the clinical relevance of circulating CXCL12α concentrations by including patients from a second PH centre (Table 1 - Sheffield cohort). CXCL12α concentrations in plasma samples collected in Sheffield independently confirmed our finding in the Dublin cohort; CXCL12α concentrations were significantly elevated in patients with IPAH and CTD-PAH (Fig 2 and Table B in S1 File). No significant differences between CXCL12α concentrations in PAH and CTD were observed between the Dublin and Sheffield cohorts (Figs 1 and 2). Since our patient cohort consisted of both incident and prevalent patients, we first examined if receiving specific PAH therapy had an effect on CXCL12α concentration. We observed no significant difference in CXCL12α concentrations between treatment naïve patients, with a median (lower-upper quartile) of 2928 pg/ml (2349–3306 pg/ml) and those on specific PAH therapy (2613 pg/ml (2208–3085 pg/ml). Since PAH is a rare disease, the study cohorts were amalgamated in all subsequent analyses. Using ROC curve analysis we observed that plasma CXCL12α concentrations distinguished between healthy (n = 44) and PAH patients (n = 95) (Fig 3). We noted a positive correlation (Rho value = 0.379; p = 0.0003) between CXCL12α levels and mean right atrial pressure (mRAP) but not with any other baseline haemodynamic measurements (Table 2).

**Table 2:** Relationship between CXCL12α concentrations and clinical indices of disease severity in PAH patients (n = 95).

Clinical Variable		CXCL12α (pg/ml) < Median#	CXCL12α (pg/ml) > Median#	Correlations (Rho)	P Value
**Age (Yrs)**		63 (47–70)	61 (47–69)	-0.045	0.665
**Gender (F:M)**		35:13	30:17		0.341
**mPAP (mmHg)**		46 (34–55)	50 (38–60)	0.152	0.149
**PCWP (mmHg)**		10 (7–12)	11 (8–14)	0.162	0.132
**mRAP (mmHg)**		7 (4–9)	10 (7–14)	0.379	0.0003*
**PVR, dyn.s/cm** ^**5**^		539 (379–800)	752 (344–1085)	0.111	0.332
**CO (L/min)**		4.9 (4.2–5.7)	4.5 (3.6–5.9)	-0.071	0.548
**WHO FC**	I	2	0		0.566
	II	11	12		
	III	31	31		
	IV	4	4		
**ISWT (m)**		195 (93–270)	120 (70–230)	-0.183	0.213
**6MWT (m)**		410 (314–475)	408 (281–450)	-0.048	0.744

Data are presented as median (lower-upper quartile)

*P<0.05 was considered statistically significant.

Definition of abbreviations: mPAP = Mean Pulmonary Artery Pressure, PCWP = Pulmonary Capillary Wedge Pressure, mRAP = Mean Right Atrial Pressure, PVR = pulmonary vascular resistance, CO = Cardiac output, WHO-FC = World Health Organization functional class, ISWT = Incremental Shuttle Walk Test (Sheffield only) and 6MWT = Six minute walk test (Dublin only).

**#**median value = 2,838 pg/ml

### Survival analysis in the combined Dublin and Sheffield cohorts

**Fig 4:**
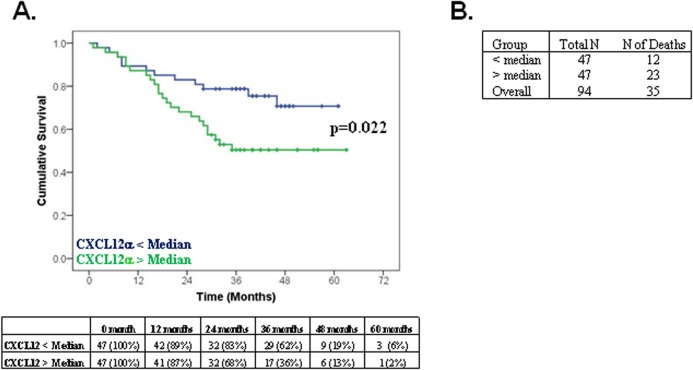
CXCL12α concentrations and survival in the combined Dublin and Sheffield PAH patients. **(A)** Kaplan Meier survival analysis among PAH patients (IPAH and CTD-PAH) with CXCL12α concentrations less than the median (blue line) and greater than the median (green line). The table represents the number of patients remaining (did not die/were not censored) in the study at the indicated time point. Note that this analysis was carried out on 94 of 95 patients; 1 patient was lost to follow-up due to emigration. **(B)** Number of deaths in entire cohort.

During the 63-month period of follow-up in Dublin, 11 of the 42 patients for whom we had follow-up data died whereas 24 of the 52 patients died during the 57-month period of follow-up in Sheffield. Kaplan-Meier analysis of cumulative survival indicated that patients with higher CXCL12α concentrations (> group median of 2,841 pg/ml) had a significantly poorer survival over time compared to patients with a lower plasma CXCL12α (< group median) (Fig 4A; p = 0.022) over the course of the study; 49% of patients with high plasma CXCL12α died compared to 26% of those with low plasma CXCL12α (Fig 4B). Interestingly, the median CXCL12α concentration in the patient group identified non-disease controls with high specificity (i.e. approx. 97.5%); this suggested those PAH patients whose CXCL12α concentration lie above normal range have a poorer prognosis.

Univariate analysis of risk factors for death revealed that high plasma CXCL12α levels, male gender, high WHO functional class, older age and incident disease were significantly associated with a shorter survival time (Table 3). Multivariate analysis showed that elevated CXCL12α was an independent risk factor (P = 0.047) for reduced survival in PAH patients, increasing the hazard ratio by 2.3-fold (Table 3). In addition, male gender (P = 0.003) and high WHO functional class (P = 0.009) were also independent risk factors for reduced survival in PAH (Table 3).

**Table 3:** Univariate and multivariate Cox proportional hazards model analysis of survival in PAH patients (n = 94).

	Alive [n (%)]	Dead [n (%)]	UnivariateP value	UnivariateHazard Ratio (95% CI)	MultivariateP value	MultivariateHazard Ratio (95% CI)
**CXCL12α concentration**						
Low CXCL12α	35 (74%)	12 (26%)	0.026	2.22 (1.10–4.47)	0.047*	2.25 (1.01–5.00)
High CXCL12α	24 (51%)	23 (49%)				
**Gender**						
Female	47 (73%)	17 (27%)	0.001	3.01 (1.55–5.87)	0.003*	2.82 (1.42–5.59)
Male	12 (40%)	18 (60%)				
**WHO_FC**						
Class 1 & 2	23 (96%)	1 (4%)	0.006	16.03 (2.19–117.35)	0.009*	15.44 (2.0–119.2)
Class 3 & 4	36 (51%)	34 (49%)				
**Treatment status**						
Prevalent	26 (76%)	8 (24%)	0.045	2.25 (1.02–4.95)	0.628	0.79 (0.31–2.02)
Incident	33 (55%)	27 (45%)				
**Disease Aetiology**	** **	** **	** **	** **		
IPAH	33 (72%)	13 (28%)	0.092	1.81 (0.91–3.60)	0.180	1.61 (0.80–3.25)
CTD	26 (54%)	22 (46%)				
**Age**						
Low Age	39 (75%)	13 (25%)	0.010	2.46 (1.24–4.88)	-	-
High Age	20 (48%)	22 (52%)				

Selected variables with p<0.10 in univariate analysis were included in the multivariate (MV) analysis

*p<0.05 considered significant in MV analysis.

Definition of abbreviations: IPAH = Idiopathic pulmonary arterial hypertension, CTD = Connective tissue disease, WHO FC = World health organization functional class. Low CXCL12α <Median (2,841 pg/ml), High CXCL12α >Median (2,841 pg/ml). Low Age<Median (63 yrs), High Age>Median (63 yrs). Prevalent: Receiving PAH therapy; Incident: Treatment naïve.

### Survival analysis in WHO functional class 3 patients

**Fig 5:**
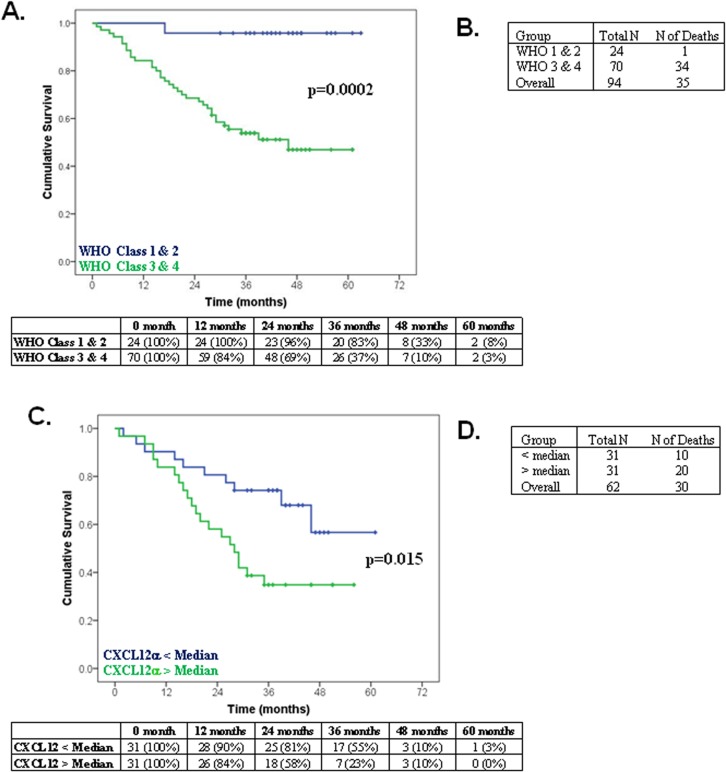
CXCL12α concentrations and survival in the combined Dublin and Sheffield PAH patients (IPAH and CTD-PAH) in WHO functional class 3. **(A)** Kaplan Meier analysis confirming that WHO functional class is strongly predictive of mortality (p = 0.0002) in PAH patients (IPAH and CTD-PAH). The table represents the number of patients remaining (did not die/were not censored) in the study at the indicated time point. **(B)** Number of patients that died in the different WHO functional classes. **(C)** Stratification of functional class 3 PAH patients (p = 0.015) on CXCL12α concentrations less than (blue line) and greater than the median (green line). The table represents the number of patients remaining (did not die/were not censored) in the study at the indicated time point. **(D)** Number of deaths in WHO functional class 3.

We confirmed that the well-established risk factor for reduced survival in PAH, namely elevated WHO-FC, was strongly associated with reduced survival (Fig 5A; P = 0.0002); PAH patients in a combined WHO functional class 1 & 2 had a significantly better survival (i.e. 4% of patients died) compared to those in WHO functional class 3 & 4 (i.e. 49% of patients died; Fig 5B). Moreover, of those patients in the combined WHO functional class 3 & 4 group, 62 were in WHO functional class 3 and 30 (48%) of these died during the course of the study. In order to determine whether CXCL12α concentrations could discriminate between those with a good or poor prognosis in this the largest group by functional class, we next examined whether elevated plasma CXCL12α concentrations could predict reduced survival in WHO functional class 3 patients. We found that patients with high CXCL12α had a significantly poorer survival rate when compared to those with low CXCL12α (Fig 5C; P = 0.015); that is to say that 65% of patients with high CXCL12α died over the course of the study whereas only 32% of patients with a low CXCL12α died (Fig 5D).

Analysis of risk factors for survival in WHO-FC 3 patients revealed that, similar to the entire PAH group, elevated CXCL12α was an independent risk factor for survival (P = 0.037), increasing the hazard ratio by more than twofold (Table 4). In addition, male gender (P = 0.008) was also an independent risk factor for reduced survival (Table 4).

**Table 4:** Univariate and multivariate Cox proportional hazards model analysis of survival in PAH patients categorised as WHO-functional class 3 (n = 62).

	Alive [n (%)]	Dead [n (%)]	UnivariateP value	UnivariateHazard Ratio (95% C.I.)	MultivariateP value	MultivariateHazard Ratio (95% C.I.)
**CXCL12α concentration**						
Low CXCL12α	21 (68%)	10 (32%)	0.019	2.50 (1.17–5.36)	0.037*	2.27 (1.05–4.89)
High CXCL12α	11 (35%)	20 (65%)				
**Gender**						
Female	27 (63%)	16 (37%)	0.004	2.93 (1.42–6.06)	0.008*	2.68 (1.29–5.56)
Male	5 (26%)	14 (74%)				
**Treatment status**						
Prevalent	10 (63%)	6 (37%)	0.313	1.59 (0.65–3.89)	-	-
Incident	22 (48%)	24 (52%)				
**Disease Aetiology**	** **	** **	** **	** **	** **	** **
IPAH	15 (54%)	13 (46%)	0.867	1.06 (0.51–2.20)	-	-
CTD	17 (50%)	17 (50%)				
**Age**						
Low Age	10 (100%)	-	0.087	2.20 (0.89–5.43)	-	-
High Age	12 (28%)	30 (72%)				

Selected variables with p<0.10 in univariate analysis was included in the multivariate (MV) analysis

*p<0.05 considered significant in MV analysis.

Definition of abbreviations: IPAH = Idiopathic pulmonary arterial hypertension, CTD = Connective tissue disease, WHO FC = World health organization functional class. Low CXCL12α <Median (2,841 pg/ml), High CXCL12α >Median (2,841 pg/ml). Low Age<Median (63 yrs), High Age>Median (63 yrs). Prevalent: On PAH therapy; Incident: Treatment naïve.

## Discussion

We have demonstrated for the first time, in a cohort of patients with pulmonary arterial hypertension, that plasma concentrations of CXCL12α are elevated compared to normal controls. In addition, we have demonstrated that elevated levels of CXCL12α are independently associated with reduced survival, and that higher CXCL12α concentration is an independent risk factor for an earlier death.

CXCL12 is a potent pro-angiogenic chemokine that signals via two receptors, CXCR4 and CXCR7 [16, 17]. Previous evidence from studies in rodents had demonstrated that CXCL12, acting through both these receptors, plays an important role in the pathogenesis of PH [8–12]. Since CXCL12 is a secreted chemokine, we hypothesised that plasma CXCL12 concentrations would be elevated in patients with PAH and would predict a poorer outcome. To examine this premise, we measured plasma CXCL12α concentrations in PAH patients attending the MMUH pulmonary hypertension clinic in Dublin. Initial findings of significantly higher CXCL12α concentrations in PH patients compared to age and gender matched controls (Fig 1) supported this hypothesis and were subsequently confirmed in a second cohort of patients recruited in Sheffield (Fig 2). These findings contrast with previous reports that plasma CXCL12α was not significantly elevated in PAH patients [18, 19]. One striking difference between patients in the current study and those in the Farha *et al* study [18] that may explain the difference in findings is that a substantial majority of the latter patients (58%) were being treated with prostacyclin analogues whereas only 12% of those we studied (11 of 95 patients) were receiving such agents (Table 1). Prostacyclin agonists potently suppress cytokine secretion by inhibition of NFκB mediated gene expression both *in vitro* and in pulmonary hypertensive patients [20, 21]. Since CXCL12 is an NFκB regulated chemokine [22], prostacyclin therapy in PH may suppress plasma CXCL12 concentrations, thus accounting for the absence of elevation of CXCL12 in patient groups where a high proportion were receiving such treatment [18]. However, direct experimental evidence in support of this proposed mechanism requires future study. Nonetheless, further evidence supporting the finding of elevated CXCL12α concentrations in PAH was provided by ROC curve analysis, which showed that plasma CXCL12α concentrations could discriminate between healthy controls and PAH patients (Fig 3).

We had initially hypothesised that if elevated CXCL12 played a significant mechanistic role in the pathogenic process underlying PH, then higher CXCL12 concentrations would predict poorer disease outcome. It was therefore interesting to note that all of our controls, with the exception of one, had a CXCL12α concentration below the median value of the patient groups. This suggests that PAH patients whose CXCL12α concentration lies above the range observed in healthy controls might have a poorer prognosis. Indeed, initial Kaplan-Meier survival analysis clearly demonstrated that high circulating CXCL12α significantly reduced survival (Fig 4). In agreement with previously published results, multivariate Cox proportional hazards model (Table 3) demonstrated an increased hazard associated with male gender and high WHO-functional class [14]. In addition to these recognised confounding factors, we now report that elevated circulating CXCL12α is an independent risk factor for survival that could potentially be included in a prognostic model. Taken together with previous evidence from animal studies that blockade of CXCL12α or its cognate receptors ameliorate the development of PAH [8–12], our finding that high circulating CXCL12α concentrations predict a poorer outcome in patients with PH suggests that CXCL12α is an important pathogenic mediator of this disease process.

CXCL12α correlated significantly with mean right atrial pressure (mRAP) in patients (Table 2); mRAP is strongly associated with reduced survival in PAH [23]. However, CXCL12α was not significantly correlated with any other haemodynamic variable (Table 2). This lack of correlation of circulating cytokines (or other circulating factors) that predict survival outcome in PAH, with haemodynamic variables, is a well-established observation. For example, in a study of five biomarkers in PAH, Rhodes *et al* found that red cell distribution width (RDW) was not significantly correlated with any haemodynamic variable, even though it was the best in distinguishing patients with a higher risk of death. Moreover, RDW was the only marker, other than N-terminal pro-brain natriuretic peptide (NT-proBNP), which added independent predictive value in a Cox’s multivariate regression analysis [24]. Similarly, in a more recent study, the same group found that reduced microRNA–150 was an independent predictor of poor survival but was not significantly correlated with pulmonary arterial pressure, pulmonary vascular resistance, cardiac output or right atrial pressure [13]. The reasons for such lack of correlation with haemodynamic variables is unknown but suggests that such changes in peripheral blood indices may be due to mechanisms that that are independent of right ventricular compromise.

It is well established that WHO functional class is an excellent predictor of outcome in PAH [25], an observation that was replicated in the present study (Fig 5A and 5B). Functional class is an important factor in the choice of PAH therapy [26] but the clinical usefulness of functional class both for establishing prognosis and guiding therapy is limited because the great majority of patients diagnosed with PAH are in functional class III and the overwhelming majority of early deaths are in this group [13, 23, 27, 28]. When we examined whether CXCL12α concentrations could discriminate between those with a good or those with a poor prognosis within functional class III, we found a strikingly worse outcome in those with higher plasma CXCL12α concentrations (Fig 5C and 5D). What is particularly noteworthy is that multivariate Cox proportional hazards model showed that elevated CXCL12α was an independent risk factor for death in WHO functional class 3 PAH patients (Table 4). These data demonstrate that measurement of plasma CXCL12α concentrations gives prognostic information additional to that provided by functional classification, further supporting the hypothesis that increased CXCL12α may be an important pathogenic mediator in the development and progression of PAH and may provide useful information to guide therapy.

It is important to acknowledge a number of limitations of our study. Since PAH is a rare disease the number of patients in our study are restricted and further independent studies in other patient centres will be needed to confirm our finding that elevated circulating CXCL12α is independently associated with a poorer prognosis in PAH. All patients included in our study had been referred to regional centres with a specific expertise in the treatment of PAH. Given the difficulty of diagnosing this condition, these represent a cohort of patients at a later stage in the natural history of the condition with more severe disease. Therefore our findings may not apply to patients at the earliest stages in the disease course. We also noted that the gender distribution and median age in IPAH patients were different in the two PH centres; IPAH patients from Sheffield were predominantly male and older when compared to those from Dublin. A further limitation of our study is that patients were predominantly Caucasian adults and therefore our findings may not necessarily generalise to other ethnic groups and to children and young adults with this condition. Additional studies are required to determine the usefulness of plasma CXCL12α concentrations as an independent risk factor for reduced survival in those other ethnic groups and age groups.

The question that then arises is how might elevated CXCL12α be causing increased mortality? Signalling via CXCL12 has been shown to be involved in several processes implicated in the development of PH, such as disordered endothelial cell proliferation, inflammation and progenitor cell recruitment. Expression of CXCL12 is markedly increased in the pulmonary vascular endothelium and the endothelium of the vasa vasorum of larger pulmonary vessels removed from patients with PH when compared to control lungs [6, 18, 19] and endothelial cells isolated from the lungs of PAH patients produce greater amounts of CXCL12 *in vitro* than cells from control lungs [18]. Furthermore, the expression of both CXCL12 receptors, CXCR4 and CXCR7, is increased in the vascular endothelium of lungs from PAH patients [6, 19]. CXCL12 acts via the CXCR7 receptor to stimulate microvascular endothelial cell proliferation and via CXCR4 to mediate chemotactic endothelial cell migration [6]. CXCL12 is also pro-inflammatory and is a potent chemoattractant for T-lymphocytes and monocytes *in vitro* [29] and stimulates progenitor cell recruitment to the pulmonary vasculature [8, 10, 11, 30]. Importantly blockade of CXCR4 or CXCR7 attenuates the development of hypoxic PH in *in vivo* murine studies [8, 10, 11] and antagonism of CXCR7 has recently been reported to ameliorate PH in new-born mice exposed to hypoxia for two weeks [9]. Taken together with our findings in patients with PAH, these data strongly suggest an important pathogenic role for elevated CXCL12 in the development of the vascular abnormalities underlying human PH. However it is important to stress that although we have identified an independent association between elevated plasma CXCL12α concentrations and reduced survival, causality cannot be assumed as it is also possible that elevated CXCL12α is a reflection of more severe disease; further work is needed to investigate this possibility.

In summary, we found that plasma CXCL12α concentrations were elevated in patients with pulmonary hypertension compared to healthy controls. We further showed that elevated circulating CXCL12α is an independent risk factor for reduced survival that could potentially be included in a prognostic model and guide therapy in clinical practice. Additional studies in larger patient cohorts, to establish the reliability and overall usefulness of circulating CXCL12α levels in predicting survival, are now required.

## Supporting Information

S1 File Supplemental methods on patient recruitment and sample processing. Table A in S1 File. Age and gender matched study participants from Dublin. This table reports the age, gender and ethnicity of the subsets of patients (n = 20 per groups) with IPAH and CTD-PAH from the Dublin cohort, together with matched controls, used to measure plasma CXCL12 concentrations. These subjects CXCL12 concentrations are graphed in Fig 1. Data are presented as median (lower-upper quartile). Table B in S1 File. Age and gender matched study participants from Sheffield. This table reports the age, gender and ethnicity of the subsets of patients (n = 19 or 21 per groups) with IPAH and CTD-PAH from the Sheffield cohort, together with matched controls from Dublin, used to measure plasma CXCL12 concentrations. These subjects CXCL12 concentrations are graphed in Fig 2. Data are presented as median (lower-upper quartile).
(DOCX)
