# Supplementary material for: Elevated Plasma CXCL12α Is Associated with a Poorer Prognosis in Pulmonary Arterial Hypertension
Source: PLoS One. 2015 Apr 9;10(4):e0123709. doi: 10.1371/journal.pone.0123709 (PMC4391833; doi:10.1371/journal.pone.0123709)
Supplement: S1 File — Table A in S1 File. Age and gender matched study participants from Dublin. This table reports the age, gender and ethnicity of the subsets of patients (n = 20 per groups) with IPAH and CTD-PAH from the Dublin cohort, together with matched controls, used to measure plasma CXCL12 concentrations. These subjects CXCL12 concentrations are graphed in Fig 1. Data are presented as median (lower-upper quartile). Table B in S1 File. Age and gender matched study participants from Sheffield. This table reports the age, gender and ethnicity of the subsets of patients (n = 19 or 21 per groups) with IPAH and CTD-PAH from the Sheffield cohort, together with matched controls from Dublin, used to measure plasma CXCL12 concentrations. These subjects CXCL12 concentrations are graphed in Fig 2. Data are presented as median (lower-upper quartile). (DOCX) [file pone.0123709.s001.docx]

**SI Supplemental Information**

**Elevated plasma CXCL12 is associated with a poorer prognosis in pulmonary arterial hypertension**

Brian N. McCullagh^1*^, Christine M. Costello^1*^, Lili Li^1^, Caroline O’Connell^1,6^, Mary Codd^2^, Allan Lawrie^3^, Allison Morton^4^ David G Kiely^5^, Robin Condliffe^5^, Charles Elliot^5^, Paul McLoughlin^1#^, Sean Gaine^6^.

**Affiliations:** ^1^School of Medicine and Medical Science, UCD Conway Institute, University College Dublin, Dublin 4, Ireland, ^2^School of Public Health, Physiotherapy and Population Science, University College Dublin, Dublin 4, Ireland; ^3^Department of Cardiovascular Sciences, University of Sheffield, Sheffield, United Kingdom ^4^Department of Cardiology, Northern General Hospital, Sheffield, United Kingdom, ^5^Sheffield Pulmonary Vascular Disease Unit, Royal Hallamshire Hospital, Sheffield, United Kingdom; ^6^Pulmonary Hypertension Unit, Mater Misericordiae University Hospital, Dublin 7, Ireland.

* These authors contributed equally to this work.

**Supplemental Information**

**Patient Recruitment**

All patients recruited to the study had a diagnosis of PH confirmed by right heart catheterization demonstrating a mean pulmonary arterial pressure greater than 25 mmHg together with a pulmonary capillary wedge pressure less than or equal to 15 mmHg. IPAH patients had pulmonary hypertension in the absence of an identifiable risk factor and without a prior family history. CTD-PAH patients had PH with evidence of systemic sclerosis (n=19), mixed connective tissue disease, including Sjogrens and systemic lupus erythematosis (n=3) or rheumatoid arthritis (n=1). Patients were diagnosed with systemic sclerosis by relevant findings from history and examination including the presence of thickened, hardened skin of the fingers, hands and face, Raynaud’s phenomenon and telangiectasia with or without extra skin manifestations and positive serology for anti-nuclear antibodies ([1](#_ENREF_1), [2](#_ENREF_2)) Sjogren’s syndrome was diagnosed by the presence of dry eyes and mouth with anti Ro/SSA and anti La/SSB antibodies in serum ([3](#_ENREF_3), [4](#_ENREF_4)). Systemic lupus erythematosis was diagnosed by a compatible history of disease and the presence of anti-double stranded DNA, anti-smith or anti-histone antibodies ([5](#_ENREF_5)). Diagnosis of rheumatoid arthritis was made on an aggregation of characteristic symptoms, signs, laboratory tests and x-ray findings, including symmetrical peripheral polyarthropathy, morning stiffness, rheumatoid factor positivity and bony erosions on x-ray ([6](#_ENREF_6)).

**Sample Porcessing**

**Pilot study**

In Dublin, venous blood samples were drawn, anti-coagulated with lithium heparin and the plasma separated by centrifugation at 1,000g for 10 minutes at 4˚C. In Sheffield, venous blood was anti-coagulated using EDTA and the plasma separated by centrifugation at 3,000g for 30 minutes at 4°C. Since plasma samples from Dublin and Sheffield were processed differently, we examined whether the anti-coagulation method or centrifugation had any effect on CXCL12 concentrations. Two tubes of blood were taken from subjects included in this pilot study; one was anti-coagulated with lithium heparin and the second tube was anti-coagulated with EDTA; and centrifuged as outlined above. Results from this pilot study indicated that the different processing techniques had no effect on CXCL12α concentrations (data not shown).

**Control of interassay variation**

Plasma samples from four PAH patients were assessed on all plates. The average value for each plasma sample across multiple ELISA plates was obtained and considered the “correct” value for that sample. The CXCL12α value of that sample on a single ELISA plate (batch assay) was divided by the “correct” value to yield a correction factor. Thus, for each batch, four separate factors were determined and the average of these used as a correction factor for that batch.

**Table A. Age and gender matched study participants from Dublin**

| **Dublin Cohort** | **Control group 1** | **IPAH** | **Control group 2** | **CTD-PAH** |
| --- | --- | --- | --- | --- |
| **Age (Yrs)** | 49 (38-62) | 49 (39-62) | 63 (51-70) | 63 (56-67) |
| **Gender (F:M)** | 16:4 | 16:4 | 16:4 | 16:4 |
| **Ethnicity** |  |  |  |  |
| Caucasian | 20 | 18 | 20 | 20 |
| Asian | - | 2 | - | - |

This table reports the age, gender and ethnicity of the subsets of patients (n=20 per groups) with IPAH and CTD-PAH from the Dublin cohort, together with matched controls, used to measure plasma CXCL12α concentrations. These subjects CXCL12α concentrations are graphed in Fig. 1.

Definition of abbreviations: IPAH = Idiopathic Pulmonary Arterial Hypertension, CTD = Connective Tissue Disease. Data are presented as median (lower-upper quartile).

**Table B. Age and gender matched study participants from Sheffield**

| **Sheffield Cohort** | **Control group 1** | **IPAH** | **Control group 2** | **CTD-PAH** |
| --- | --- | --- | --- | --- |
| **Age (Yrs)** | 51 (35-63) | 52 (34-66) | 61 (51-66) | 63 (61-71) |
| **Gender (F:M)** | 14:5 | 13:6 | 17:4 | 16:5 |
| **Ethnicity** |  |  |  |  |
| Caucasian | 19 | 15 | 21 | 21 |
| Asian | - | 4 | - | - |

This table reports the age, gender and ethnicity of the subsets of patients (n=19 or 21 per groups) with IPAH and CTD-PAH from the Sheffield cohort, together with matched controls from Dublin, used to measure plasma CXCL12α concentrations. These subjects CXCL12α concentrations are graphed in Figure 2.

Definition of abbreviations: IPAH = Idiopathic Pulmonary Arterial Hypertension, CTD = Connective Tissue Disease. Data are presented as median (interquartile range).

**References**

1. Lonzetti LS, Joyal F, Raynauld JP, Roussin A, Goulet JR, Rich E, et al. Updating the American College of Rheumatology preliminary classification criteria for systemic sclerosis: addition of severe nailfold capillaroscopy abnormalities markedly increases the sensitivity for limited scleroderma. Arthritis Rheum. 2001;44(3):735-6. Epub 2001/03/27.

2. Silman AJ. Scleroderma. Bailliere's clinical rheumatology. 1995;9(3):471-82. Epub 1995/08/01.

3. Vitali C. Classification criteria for Sjogren's syndrome. Annals of the rheumatic diseases. 2003;62(1):94-5; author reply 5. Epub 2002/12/14.

4. Vitali C, Bombardieri S, Jonsson R, Moutsopoulos HM, Alexander EL, Carsons SE, et al. Classification criteria for Sjogren's syndrome: a revised version of the European criteria proposed by the American-European Consensus Group. Annals of the rheumatic diseases. 2002;61(6):554-8. Epub 2002/05/15.

5. Boumpas DT, Fessler BJ, Austin HA, 3rd, Balow JE, Klippel JH, Lockshin MD. Systemic lupus erythematosus: emerging concepts. Part 2: Dermatologic and joint disease, the antiphospholipid antibody syndrome, pregnancy and hormonal therapy, morbidity and mortality, and pathogenesis. Annals of internal medicine. 1995;123(1):42-53. Epub 1995/07/01.

6. Aletaha D, Neogi T, Silman AJ, Funovits J, Felson DT, Bingham CO, 3rd, et al. 2010 Rheumatoid arthritis classification criteria: an American College of Rheumatology/European League Against Rheumatism collaborative initiative. Arthritis Rheum. 2010;62(9):2569-81. Epub 2010/09/28.
